# Functional dissection of two amino acid substitutions unique to the human FOXP2 protein

**DOI:** 10.1038/s41598-023-30663-3

**Published:** 2023-03-06

**Authors:** Ulrich Bornschein, Hugo Zeberg, Wolfgang Enard, Wulf Hevers, Svante Pääbo

**Affiliations:** 1 Max Planck Institute for Evolutionary Anthropology, Deutscher Platz 6, 04103 Leipzig, Germany; 2 Department of Pharmacology and Physiology, Karolinska Institutet, 17177 Stockholm, Sweden; 3 Okinawa Institute of Science and Technology, Onna-Son, Japan; 4 Present Address: Faculty of Biology, Ludwig Maximilian University, 82152 Martinsried, Germany

**Keywords:** Evolutionary genetics, Development of the nervous system

## Abstract

The transcription factor forkhead box P2 (FOXP2) is involved in the development of language and speech in humans. Two amino acid substitutions (T303N, N325S) occurred in the human FOXP2 after the divergence from the chimpanzee lineage. It has previously been shown that when they are introduced into the FOXP2 protein of mice they alter striatal synaptic plasticity by increasing long-term depression in medium spiny neurons. Here we introduce each of these amino acid substitutions individually into mice and analyze their effects in the striatum. We find that long-term depression in medium spiny neurons is increased in mice carrying only the T303N substitution to the same extent as in mice carrying both amino acid substitutions. In contrast, the N325S substitution has no discernable effects.

## Introduction

**Figure 1:**
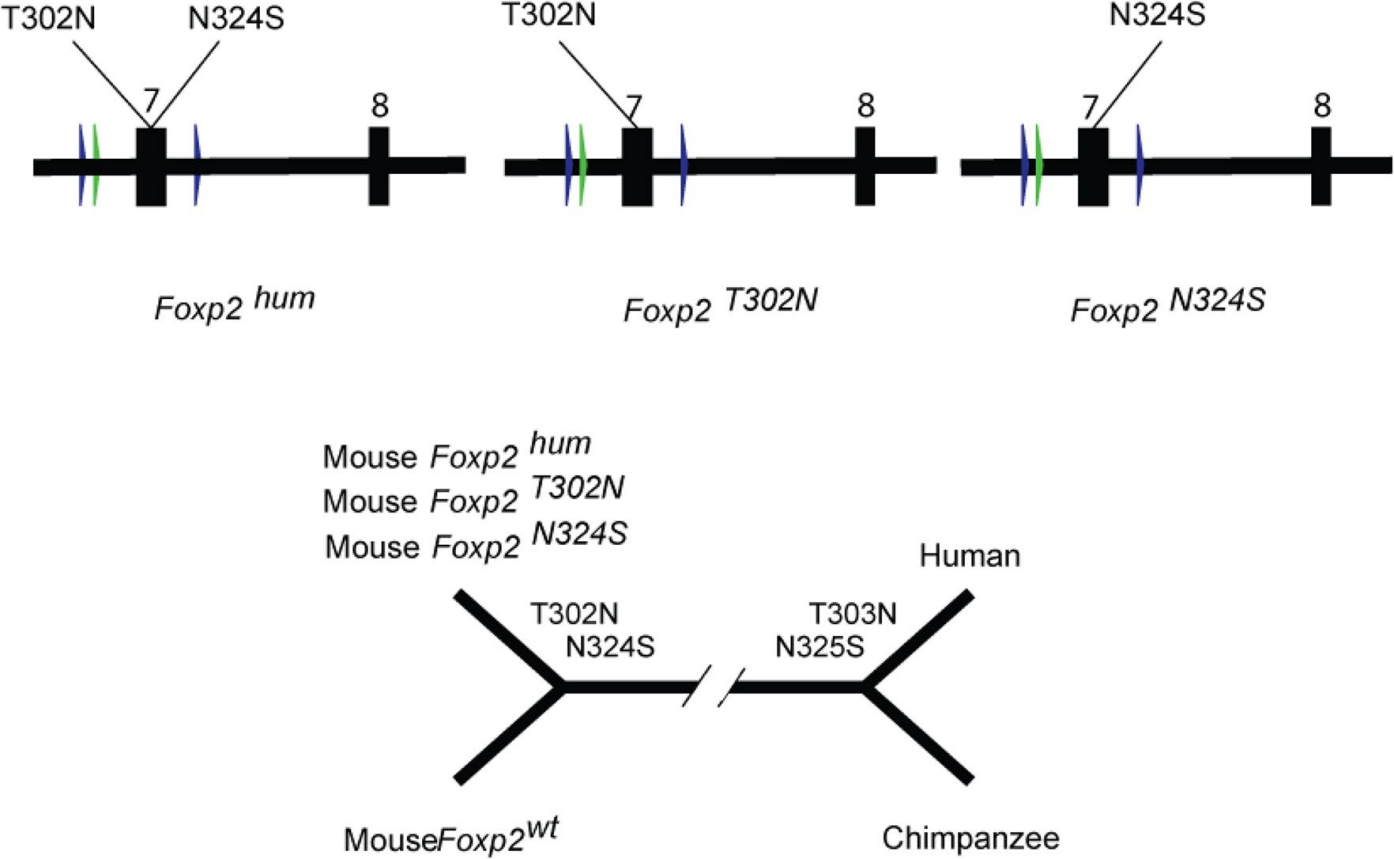
Overview of experimental setup. Blue arrows: loxP sites; green arrow: remaining FRT site.

Humans heterozygous for pathogenetic variants of *FOXP2* have difficulties in learning and performing complex orofacial movements, including those used for speech, as well as deficits in receptive and expressive language^1–3^. Although the transcription factor FOXP2 is thus involved in a trait unique to humans, only three out of the 715 amino acids in the FOXP2 protein differ between humans and mice, making it among the 5% most conserved proteins between the two species^4^. Remarkably, two of these amino acid substitutions (T303N and N325S) occurred on the human evolutionary lineage since its divergence from the chimpanzee lineage (Fig. 1). They have therefore been suggested to be of relevance for the evolution of speech and language^4–6^. Mice where these two human substitutions have been introduced into the endogenous *Foxp2* gene (*Foxp2*^*hum*^ mice) are generally healthy but show enhanced synaptic plasticity in the form of stronger long-term depression (LTD) in the medium spiny neurons (MSN) of the striatum, suggesting that cortico-basal ganglia circuits are affected^7–9^. However, it is unclear whether one or both of the two amino acid changes cause these effects.

To better understand the contributions of the two amino acid substitutions, we have generated one mouse line which carries only the threonine-to-asparagine substitution at position 302 (T302N; N303 in humans) and one mouse line which carries only the asparagine-to-serine at position 324 (N324S; S325 in humans) in their endogenous *Foxp2* genes. Here, we analyze how these *Foxp2* alleles (*Foxp2*^*T302N*^, *Foxp2*^*N324S*^) affect LTD in MSNs.

## Results

### Generation of *Foxp2* mice

Mice carrying *Foxp2* alleles encoding each of the two single amino acid substitutions (*Foxp2*^*N324S*^, *Foxp2*^*T302N*^) were generated (Fig. 1) as previously described for the *Foxp2*^*hum*^ mice^7^. Targeted integration of the constructs and mutations was verified by Southern blot analysis and sequencing (Fig. S1). We have previously shown that the *Foxp2* gene is expressed at the RNA and protein levels in in the identically constructed *Foxp2*^*hum*^ mice (Figures S1 and S2 in^7^). We observed no loss-of-function phenotypes, such as postnatal death, weight loss or other developmental abnormalities that are characteristic for mice homozygous for null alleles of *Foxp2*^10,11^. We compared animals homozygous for each of the two *Foxp2* versions (*Foxp2*^*N324S*^, *Foxp2*^*T302N*^) with littermates or cousins homozygous for the wild type allele (*Foxp2*^*wt*^). Hence, phenotypic differences among the animals carrying the different *Foxp2* versions are likely to be caused by the amino acid differences in Foxp2 (Fig. 1).

### Synaptic plasticity in ***Foxp2***^***T302N***^ and ***Foxp2***^***N324S***^ mice

**Figure 2:**
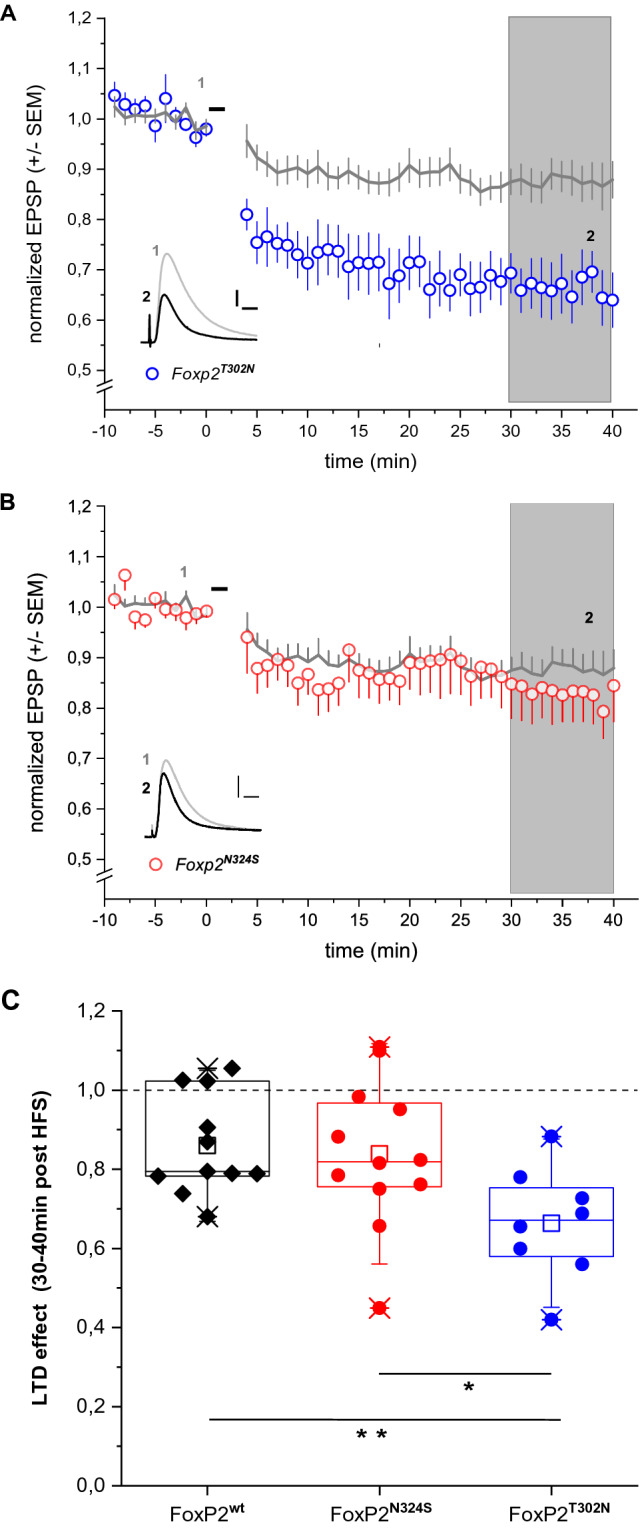
Effects of human amino acid substitutions in FOXP2 on synaptic plasticity in striatal neurons in (**A**) *Foxp2*^*T302N*^ mice and (**B**) *Foxp2*^*N324S*^ mice. LTD was induced in MSN by high-frequency stimulation (100 Hz, black bars; see “Experimental procedures”). Amplitudes normalized to baseline levels (mean ± SEM, t = 10 to 0) is shown. Insets in A and B show representative EPSP traces averaged over 5 min at time point 1 (pre high-frequency stimulation) and 2 (post high-frequency stimulation, scale bars 2 mV, 20 ms). For comparison, mean amplitudes for wild-type (*Foxp2*^*wt*^, grey line) are given in A and B. Boxplots in (**C**) illustrate the LTD effect for each strain (average reduction of EPSP amplitude t = 30–40 min compared to baseline (t = − 10 to 0 min, *P = 0,028, **P = 0,011, MWU).

We have previously shown that LTD in neurons of the central^7^ and dorsolateral parts^8,9^ of the striatum of *Foxp2*^*hum*^ mice is enhanced. Using identical protocols, we made dorsolateral recordings from *Foxp2*^*T302N*^ (N = 8) and *Foxp2*^*N324S*^ (N = 12) and their wildtype littermates (N = 3 and N = 8, respectively) and compared them to previously published data of *Foxp2*^*hum*^ mice^8,9^. At 30–40 min after high-frequency stimulation, LTD of *Foxp2*^*T302N*^ cells is increased compared to *Foxp2*^*wt*^ cells (Mann Whitney U test (MWU): *P* = 0.011, Fig. 2A,C, Supplementary Table S1). When compared to the previously published data for *Foxp2*^*hum*^ cells, no significant differences are seen (MWU: *P* = 0.48). In contrast, LTD in *Foxp2*^*N324S*^ cells differs from *Foxp2*^*T302N*^ cells (MWU: *P* = 0.028, Fig. 2C) and is not detectably different from *Foxp2*^*wt*^ cells (MWU: *P* = 0.93; Fig. 2B,C, Supplementary Table S1).

## Discussion

A question regarding the two amino acid substitutions that affected the FOXP2 protein during human evolution is if they both have physiological consequences or if only one of them has effects. Here we show that mice homozygous for the T302N substitution exhibit increased LTD in the dorsolateral striatum to an extent similar to mice carrying both amino acid substitutions. In contrast, LTD in mice homozygous for N324S substitution does not differ from LTD in their wildtype littermates (Fig. 2). Thus, whereas the T302N substitution has clear effects in terms of synaptic plasticity, the N324S substitution does not have such effects. More work is needed to understand how these observations relate to phenotypic effects that the two amino acid substitutions have in the mouse, notably subtle differences in vocalization^5^ and enhanced transition from declarative to procedural performance during certain learning tasks^9^.

However, the variation in FOXP2 amino acid sequences among and within species can give indications about how important the two amino acid substitutions may be. Among vertebrates, the N324S substitution occurs also in carnivores and birds and is thus not unique to humans. Furthermore, 26% of western gorillas carry a substitution two amino acids away at position 326 (A326S)^12^. In contrast, the T302N substitution has not been observed in any other organism than humans. Among 125,642 humans for which exome sequences are available (gnomAD, v2.1.1), no individual carry the ancestral amino acid variants at either position. However, two individuals carry a serine to arginine substitution at the position corresponding to position 324 in the mouse (S325R). Thus, whereas the T303N substitution in humans affects a position that is extremely conserved and not known to vary among humans today, the N325S substitution has occurred also among other mammals and the positions varies among humans, albeit very rarely.

It should be noted that a signature of a recent selective sweep that was originally linked to the two changes^5^ is not supported when using sequence data from hundreds of globally distributed humans^13^*.* However, the two amino acid substitutions analyzed here are shared with Neandertals and Denisovans^14^—human forms that diverged from the ancestors of present-day humans 550,000–765,000 years ago^15^. Hence, signatures of recent selection, which are extremely unlikely to be detected when they occurred that long ago, are uninformative with respect to these two amino acid changes. Nevertheless, other functional changes may have affected the *FOXP2* gene on the human evolutionary lineage more recently. For example, a substitution in a transcription factor-binding site in intron 8 of the *FOXP2* gene is unique to modern humans and thus occurred during the past 500,000 years. The transcription factor that binds to this site, POU3F2 (also called BRN2), is expressed uniquely in the nervous system and is involved in neuronal differentiation and the substitution reduces the efficiency with which POU3F2 dimers bind to the binding site and drive transcription^16^. It is thus possible that multiple changes that affect both the function of the FOXP2 protein and the expression of the *FOXP2* gene have occurred on the human evolutionary lineage. However, in terms of the effects of the protein on synaptic plasticity, the T303N rather than the N325S substitution is of importance.

## Experimental procedures

### Generation of ***Foxp2***^***N324S***^ and ***Foxp2***^***T302N***^ expressing mice

The vectors for generating the *Foxp2*^*N324S*^ and *Foxp2*^*T302N*^ allele were created by mutagenesis of the targeting vector used for the *Foxp2*^*hum*^ allele (details see^7^). Linearized, sequence verified vectors were electroporated into Bruce4 C57BL/6 ES cells by Ozgene (Bentley, Australia) as described^7^. ES clones were screened and verified for targeted integration by Southern blot analysis (Fig. S1A,B; the original blots as provided by the company Ozgene are given in S1C) and point mutations in exon 7 were confirmed by sequencing before generation of chimeras. The resulting founder mice were bred, point mutations again confirmed by sequencing (Figure S1D) and then crossed to mice transgenic for the recombinase FLPe under the control of the human *ACTB* promoter (Jackson Laboratory, Stock Number 003800; C57BL/6J^17^) to generate *Foxp2*^*N324S*^ or *Foxp2*^*T302N*^ alleles in which the FRT-flanked neomycin resistance cassettes have been removed as described^7^. The recombinase transgenes were outcrossed using C57BL/6J mice in the next generation and further crossings were made in C57BL/6J mice (C57BL/6J@Rj; Janvier, St. Berthevin, France). Hence, all alleles are on the same genomic background and lack the neomycin resistance cassette of the targeting vector. Mice used for recordings were homozygous for the wildtype (*Foxp2*^*wt*^) or *the FoxP2 locus (Foxp2*^*N324S*^ or *Foxp2*^*T302N*^). They were either derived directly from the first generation of crossings of heterozygous animals or from the following second generation where homozygous wildtype or FoxP2 siblings from the first generation were crossed. Thus, mice compared with each other were either matched littermates or second generation offsprings of such littermates (cousins). Genotyping was done as described^7^. All mouse experiments were overseen and approved by the Institutional Animal Welfare Officer of the Max Planck Institute for Evolutionary Anthropology (Dr. Gerd Möbius, Fac. of Veterinary Medicine, Univ. Leipzig). They were performed in accordance to the German Animal Welfare Legislation (“Tierschutzgesetz”), and approved and registered with the Federal State Authority Landesdirektion Sachsen (No. 24-9162.11 (T 38/12)).

We recently discovered that a wildtype deletion as described for the Harlan line C57BL/6JOlaHsd (365 kb between pos. 60.976 and 61.341 Mb of Chr. 6, including the *Snca* and *Mmrn1* locus^18,19^) occurred in some of our lines (incl. the FLPe line). Hence, we tested all animals used in these experiments with PCR protocols as given in^19^. No animals carrying this deletion were found.

### Slice electrophysiology

Brains of slightly anesthetized mice (P21–P53; isoflurane) were prepared into ice-cold sucrose-based cutting solution (in mM: 85 sucrose, 60 NaCl, 3.5 KCl, 6 MgCl_2_, 0.5 CaCl_2_, 38 NaHCO_3_, 1.25 NaH_2_PO_4_, 10 HEPES, 25 glucose). Coronal slices (250 µm) were cut (Vibroslice 7000smz, Campden Instruments, UK), incubated in artificial cerebrospinal fluid (aCSF; in mM: 120 NaCl, 3.5 KCl, 1 MgCl_2_, 2 CaCl_2_, 30 NaHCO_3_, 1.25 NaH_2_PO_4_, 15 glucose) supplemented with 5 mM HEPES, 1 MgCl_2_ for 30 min at 35 °C and allowed to recover at room temperature for at least 40 min.

MSN were identified as in^20^. They were recorded in the current clamp configuration with the bridge mode enabled (EPC-10 amplifier, Patch- and Fitmaster software; HEKA, Lambrecht, Germany). The internal solution contained (in mM): 150 K-gluconate, 10 NaCl, 3 Mg-ATP, 0.5 GTP, 10 HEPES and 0.05 EGTA adjusted to pH = 7.3 and 310 mOsm with the liquid junction potential (15 mV) corrected online. Slices were perfused (2–3 ml/min, aCSF, 21–24 °C) in presence of the GABA_A_R antagonist gabazine (SR-95531, 10 µM, Sigma). All solutions were continuously oxygenated with 95% O_2_, 5% CO_2_ gas.

Glutamatergic excitatory afferents where stimulated intrastriatally with aCSF-filled theta-glass electrodes typically ~ 100–150 µm away from the MSN soma (position of stimulation electrode between MSN and corpus callosum). A bipolar voltage pulse (0.1 ms, ± 5 to ± 30 V) at 0.2 Hz induced subthreshold excitatory postsynaptic potentials (EPSPs; 4–10 mV). Following 10–15 min baseline recording synaptic plasticity was induced by a high frequency protocol (four 100 Hz tetani, 3 s long, separated by 30 s; holding potential − 70 mV). Recordings were rejected if the membrane potential was more positive than − 80 mV or the input resistance changed by more than 30%. We verified that no background long-term potentiation was present as APV ((2*R*)-amino-5-phosphonovaleric acid), a specific blocker of a subtype of glutamate receptors, did not alter the effect in wildtype mice^9^.

### Statistical analysis

EPSP amplitudes were normalized to a mean baseline level at t = − 10 to 0 min. LTD magnitude of individual cells was calculated by averaging amplitudes 30–40 min after induction with the high frequency protocol. For comparisons we used previously published data for *Foxp2*^*wt*^ and *Foxp2*^*hum*^ obtained under identical conditions^8,9^.

All analyzed cells, their associated information (animal, age, litter) and their LTD magnitude are listed in Supplementary Table S1. Genotypes were blinded for experimenters and initial evaluation. All methods are reported in accordance to the ARRIVE guidelines.

## Supplementary Information

Supplementary Information.

## Data Availability

All analyzed cells, their associated information (animal, age, litter) and their LTD magnitude are listed in Supplementary Table S1. Additional data, including the original reports by the company OzGene on generating the mice are available from the corresponding author on request.
