# Supplementary material for: Functional dissection of two amino acid substitutions unique to the human FOXP2 protein
Source: Sci Rep. 2023 Mar 6;13:3747. doi: 10.1038/s41598-023-30663-3 (PMC9988825; doi:10.1038/s41598-023-30663-3)
Supplement: Supplementary file 1 — Supplementary Information. [file 41598_2023_30663_MOESM1_ESM.pdf]

Supplementary Material

Functional dissection of two amino acid substitutions unique to the human FOXP2 protein

Authors: Ulrich Bornschein[a], Hugo Zeberg[a,b], Wolfgang Enard[a,1], Wulf Hevers[a]* & Svante Pääbo[a,c]

[a] Max Planck Institute for Evolutionary Anthropology, Deutscher Platz 6, D-04103 Leipzig, Germany
[b] Department of Pharmacology and Physiology, Karolinska Institutet, SE-17177 Stockholm, Sweden
[c] Okinawa Institute of Science and Technology, Onna-son, Japan
[1] Present address: Faculty of Biology, Ludwig Maximilian University, D-82152 Martinsried, Germany

* Correspondence to: Wulf Hevers (wulf_hevers@eva.mpg.de).

Supplementary Figure S1

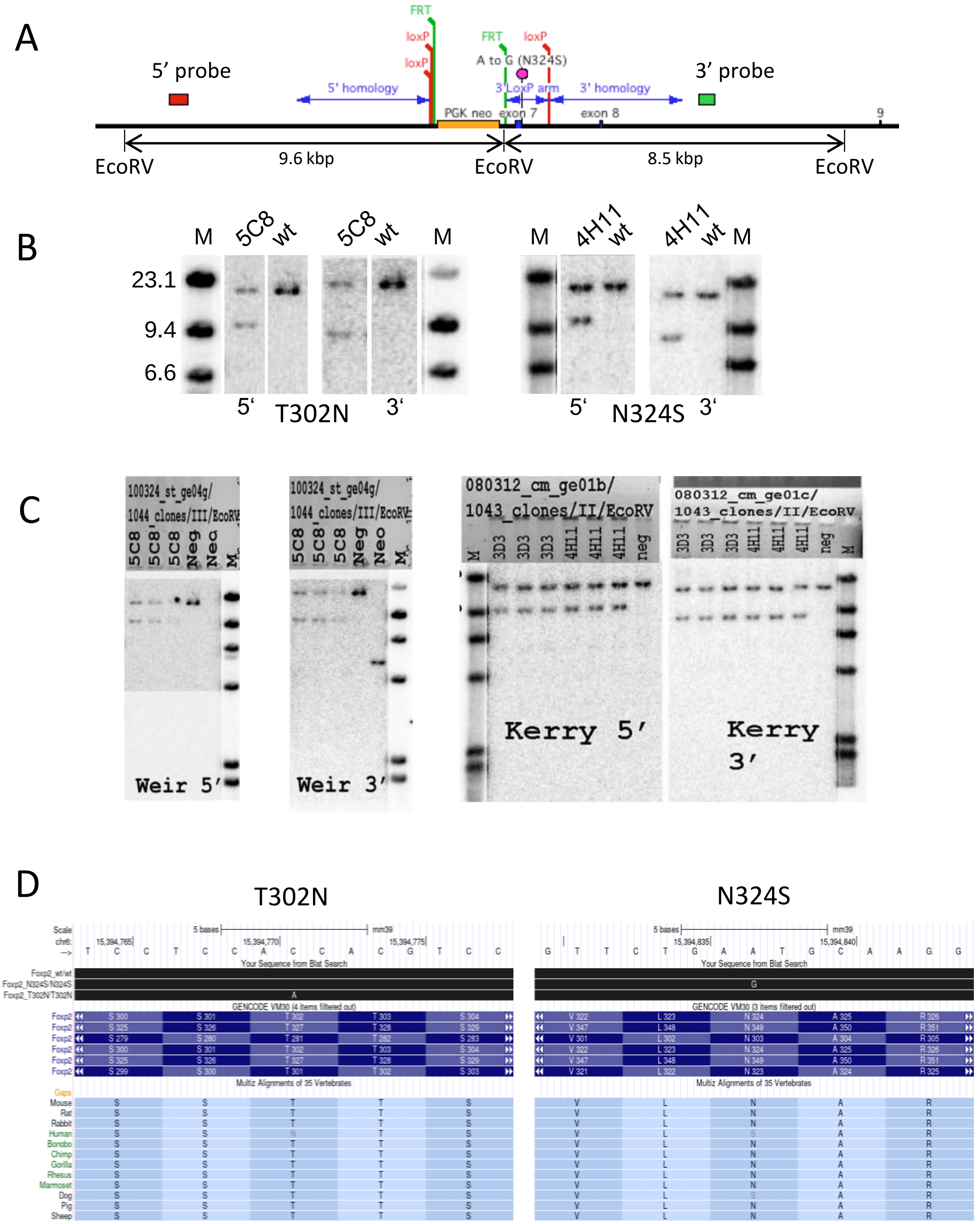

Supplementary Figure S1. Validation of targeted lines. A) Southern blot analysis of genomic DNA from the targeted mouse ES cell clones 4H11 and 5C8 that are heterozygous for the alleles *Foxp2*^{N324S} and *Foxp2*^{T302N}, respectively and were used to generate the corresponding mouse lines. **B)** EcoRV digested genomic DNA together with a radioactively labelled marker (M) was probed with the 3' probe and the 5' probe as indicated. The expected size of the *Foxp2*^{wt} allele is 16 kbp with the 3' and 5' probe. The expected sizes of both the *Foxp2*^{N324} and the *Foxp2*^{T302N} allele are 9.6 kbp and 8.5 kbp with the 5' and the 3'probe, respectively, as depicted in the targeted locus. **C)** Original blots with three replicates per clone provided in the pdf format by the company Ozgene who generated the ES clones are depicted below the cropped images. No image processing in addition to copying and cropping from pdf was done. **D)** The presence of the correct mutations was confirmed by PCR amplification the targeted exon 7 from DNA extracted from mice homozygous for *Foxp2*^{wt} (N=1), *Foxp2*^{N324} (N=3), or the *Foxp2*^{T302N} (N=2). The resulting sequences all contained the expected allelic variants as depicted on screenshots of the sequences after blating to the mouse genome (mm39; http://genome.ucsc.edu).

Supplementary Table S1

animal-ID_cell-ID	mouse line	breeding	litter_ID	age / days	genotype	effect_post_HFS
47832_1	FoxP2_new_deltaNeo	heterozygous	790_27_6	45	Foxp2_wt/wt	0,785
68173_2	Foxp2_N3245_deltaNeo	heterozygous	85_10_2	28	Foxp2_wt/wt	0,783
68174_1	Foxp2_N3245_deltaNeo	heterozygous	85_10_2	22	Foxp2_wt/wt	1,025
68176_1	Foxp2_N3245_deltaNeo	heterozygous	85_10_2	26	Foxp2_wt/wt	1,055
69025_1	Foxp2_N3245_deltaNeo	heterozygous	111_2_3	27	Foxp2_wt/wt	0,789
69025_2	Foxp2_N3245_deltaNeo	heterozygous	111_2_3	27	Foxp2_wt/wt	0,869
69025_3	Foxp2_N3245_deltaNeo	heterozygous	111_2_3	27	Foxp2_wt/wt	0,680
89829_1	FoxP2_T302_deltaNeo	homozygous	86_29_10	32	Foxp2_wt/wt	0,794
89830_1	FoxP2_T302_deltaNeo	homozygous	86_29_10	31	Foxp2_wt/wt	0,906
90782_1	Foxp2_N3245_deltaNeo	heterozygous	394_9_11	28	Foxp2_wt/wt	1,023
91050_1	FoxP2_T302_deltaNeo	homozygous	137_28_11	37	Foxp2_wt/wt	0,739
89326_1	FoxP2_T302_deltaNeo	heterozygous	85_4_10	29	Foxp2_T302N/T302N	0,727
89326_2	FoxP2_T302_deltaNeo	heterozygous	85_4_10	29	Foxp2_T302N/T302N	0,779
89480_1	FoxP2_T302_deltaNeo	heterozygous	125_13_10	23	Foxp2_T302N/T302N	0,420
89482_1	FoxP2_T302_deltaNeo	heterozygous	125_13_10	24	Foxp2_T302N/T302N	0,599
89482_2	FoxP2_T302_deltaNeo	heterozygous	125_13_10	24	Foxp2_T302N/T302N	0,560
89486_1	FoxP2_T302_deltaNeo	homozygous	102_10_10	24	Foxp2_T302N/T302N	0,688
89491_1	FoxP2_T302_deltaNeo	homozygous	87_14_10	21	Foxp2_T302N/T302N	0,882
89495_1	FoxP2_T302_deltaNeo	homozygous	87_14_10	21	Foxp2_T302N/T302N	0,655
68171_1	Foxp2_N3245_deltaNeo	heterozygous	85_10_2	27	Foxp2_N324S/N324S	0,657
68836_1	Foxp2_N3245_deltaNeo	homozygous	101_28_2	17	Foxp2_N324S/N324S	0,815
68841_1	Foxp2_N3245_deltaNeo	homozygous	101_28_2	26	Foxp2_N324S/N324S	0,951
68842_1	Foxp2_N3245_deltaNeo	homozygous	101_28_2	22	Foxp2_N324S/N324S	0,750
69026_1	Foxp2_N3245_deltaNeo	heterozygous	111_2_3	28	Foxp2_N324S/N324S	0,882
69026_2	Foxp2_N3245_deltaNeo	heterozygous	111_2_3	28	Foxp2_N324S/N324S	0,789
69026_3	Foxp2_N3245_deltaNeo	heterozygous	111_2_3	28	Foxp2_N324S/N324S	1,109
69028_1	Foxp2_N3245_deltaNeo	heterozygous	111_5_3	26	Foxp2_N324S/N324S	0,823
90781_1	Foxp2_N3245_deltaNeo	heterozygous	394_9_11	27	Foxp2_N324S/N324S	1,099
91562_2	Foxp2_N3245_deltaNeo	homozygous	402_9_12	32	Foxp2_N324S/N324S	0,449
91901_1	Foxp2_N3245_deltaNeo	homozygous	402_28_12	20	Foxp2_N324S/N324S	0,762
91901_3	Foxp2_N3245_deltaNeo	homozygous	402_28_12	20	Foxp2_N324S/N324S	0,983
47822_1	FoxP2_new_deltaNeo	heterozygous	790_22_6	43	Foxp2_hum/hum	0,334
47830_1	FoxP2_new_deltaNeo	heterozygous	790_27_6	45	Foxp2_hum/hum	0,765
47830_2	FoxP2_new_deltaNeo	heterozygous	790_27_6	45	Foxp2_hum/hum	0,227
48011_1	FoxP2_new_deltaNeo	heterozygous	793_5_7	44	Foxp2_hum/hum	1,166
48012_1	FoxP2_new_deltaNeo	heterozygous	793_5_7	44	Foxp2_hum/hum	0,384
48012_2	FoxP2_new_deltaNeo	heterozygous	793_5_7	44	Foxp2_hum/hum	0,821
48859_1	FoxP2_new_deltaNeo	heterozygous	793_10_7	53	Foxp2_hum/hum	0,872
73169_1	FoxP2_new_deltaNeo	homozygous	2327_26_6	25	Foxp2_hum/hum	0,839
73169_2	FoxP2_new_deltaNeo	homozygous	2327_26_6	25	Foxp2_hum/hum	0,852
73171_1	FoxP2_new_deltaNeo	homozygous	2327_26_6	30	Foxp2_hum/hum	0,848
73897_1	FoxP2_new_deltaNeo	homozygous	2282_18_7	17	Foxp2_hum/hum	0,735
73897_2	FoxP2_new_deltaNeo	homozygous	2282_18_7	17	Foxp2_hum/hum	0,616
73899_1	FoxP2_new_deltaNeo	homozygous	2282_18_7	24	Foxp2_hum/hum	0,609
73921_1	FoxP2_new_deltaNeo	homozygous	2327_11_7	17	Foxp2_hum/hum	0,570
74829_1	FoxP2_new_deltaNeo	homozygous	2325_11_7	43	Foxp2_hum/hum	0,971
76213_1	FoxP2_new_deltaNeo	homozygous	1891_27_8	18	Foxp2_hum/hum	0,686
76216_1	FoxP2_new_deltaNeo	homozygous	1891_27_8	19	Foxp2_hum/hum	0,735

Supplementary Table S1. Details of the animals used. Given are further data on the mice used in this study (sorted according to genotype). **animal-ID_cell-ID**: the unique animal number given by the electronic database for each animal in the facility followed by a consecutive number for the individual cell recorded from this animal. **mouse line:** the name of the mouse line as used in the electronic database. **breeding:** method used for generating the offspring using either heterozygous or homozygous parents. In the latter case the parents themselves were littermates and the offspring accordingly cousins. **litter_ID:** the litter identifier given by an identifier of the father and the birthdate. Littermates born on the same day by one pair of parents have accordingly an identical litter_ID. **age / days:** age of the animal used in days. **genotype:** genotype of the individual mouse as genotyped. **effect_post_HFS:** effect size of the LTD 30-40 min after induction, normalized to baseline levels before induction (as used in Figure 2 C; see Experimental procedures for further details).
